# Evolution in eggs and phases: experimental evolution of fecundity and reproductive timing in *Caenorhabditis elegans*

**DOI:** 10.1098/rsos.160496

**Published:** 2016-11-09

**Authors:** Bradly Alicea

**Affiliations:** 1 Orthogonal Research, Champaign, IL, USA; 2 OpenWorm Foundation, CA, USA

**Keywords:** experimental evolution, evolution of development, reproductive dynamics

## Abstract

To examine the role of natural selection in fecundity in a variety of *Caenorhabditis elegans* genetic backgrounds, we used an experimental evolution protocol to evolve 14 distinct genetic strains over 15–20 generations. We were able to generate 790 distinct genealogies, which provided information on both the effects of natural selection and the evolvability of each strain. Among these genotypes are a wild-type (N2) and a collection of mutants with targeted mutations in the *daf-c, daf-d* and AMPK pathways. Differences are observed in reproductive fitness along with related changes in reproductive timing. The majority of selective effects on fecundity occur during the first few generations of evolution, while the negative selection for reproductive timing occurs on longer time scales. In addition, positive selection on fecundity results in positive and negative strain-dependent selection on reproductive timing. A derivative of population size per generation called reproductive carry-over (RCO) may be informative in terms of developmental selection. While these findings transcend mutations in a specific gene, changes in the RCO measure may nevertheless be products of selection. In conclusion, the broader implications of these findings are discussed, particularly in the context of genotype-fitness maps and the role of uncharacterized mutations in individual variation and evolvability.

## 1. Introduction

There is a rich tradition of using the nematode *Caenorhabditis elegans* to make controlled genetic manipulations that provide significant insights regarding genetic effects on development and physiology. Experimental evolution methods provide us with the opportunity to look beyond the classical genetic methods [1], and provide us with a means to study the effects of natural selection in a way not possible with comparative or inferential techniques [2]. In particular, experimental evolution allows control and temporal resolution over the evolutionary process, which enables novel functional assessments of existing mutant genotypes [3]. Using a dataset of a single wild-type genotype and 13 mutant genotypes, we will address:

— What effect does natural selection and mutational diversity have on fecundity (as measured by population size over a finite interval)?
— Are the observed differences in population size informative with respect to genotypic identity?
— Do we observe changes in reproductive timing (as measured by reproductive carry-over (RCO)) that result from positive selection for fecundity?

Between 47 and 64 genealogies per strain have been generated for 14 genetic strains of *C. elegans* over 15–20 generations (see table 1; full information in electronic supplementary material). These genealogies result from repeated selection during each generational interval (3 day, or 6 day where noted). We terminate all non-selected replicates in every generation. Founder worms at the L4 stage of development are selected in terms of largest population sizes generated after 3 days. Founder worms for the next generation are drawn randomly from the selected populations at proportions consistent with the relative size of the selected populations. This process produces a series of new replicates representing the subsequent generation. In this way, we maintain the same number of replicates during every generation in the experiment. As we repeatedly select some populations over others, we generate a large number of genealogies of varying length.

**Table 1.** An inventory of strains and the number of genealogies generated via natural selection.

	strain (genotype identification)	no. genealogies
1	*qIs56[Plag-2::GFP]; daf-7(e1372)*	62
2	*qIs56[Plag-2::GFP]; daf-9(rh50)*	58
3	N2	62
4	*qIs56[Plag-2::GFP]; aak-1(tm1944)*	62
5	*myIs14[Pklp-6::GFP]; daf-7(e1372); daf-16(mu86)*	61
6	*qIs56[Plag-2::GFP]*	49
7	*lag-2(q420)*	64
8	*qIs56[Plag-2::GFP]; aak-2(ok524)*	50
9	*qIs56[Plag-2::GFP]; aak-1(tm1944); aak-2(ok524)*	58
10	*qIs56[Plag-2::GFP]; daf-7(e1372); daf-16(m27)*	57
11	*qIs56[Plag-2::GFP]; aak-1(tm1944); daf-7(e1372)*	47
12	*qIs56[Plag-2::GFP]; aak-2(ok524); daf-7(e1372)*	51
13	*qIs56[Plag-2::GFP]; aak-1(tm1944); aak-2(ok524); daf-7(e1372)*	53
14	*daf-16(mu86)*	56

In this study, we are not only selecting for fecundity, but in some cases also indirectly selecting for reproductive timing. While there are a finite number of eggs and sperm in each individual worm, their reproductive capacity still exhibits some variability. Some of this variability is stochastic in nature, but a major component is strongly influenced by genetic background. All things being equal in terms of environment, selecting from populations with large numbers of offspring 3 days after the founder worm's L4 stage will favour early reproducers in subsequent populations. By keeping the environment fixed across the course of our evolutionary trajectories, it is our goal to demonstrate the selective effects across a diverse range of mutant genotypes.

According to Gray & Cutter [4], life history and the effects of mutation are a fertile area for *C. elegans* experimental evolution research. In this study, we intend to take advantage of this by examining the evolvability of a variety of genetic mutant strains when positively selected for fecundity in earlier portions of the reproductive cycle. Selecting for fecundity often results in evolutionary changes over a relatively low number of generations because reproduction is sperm-limited [5]. In wild-type genetic backgrounds, life history related shifts in sperm production [6] can lead to positive selection for fecundity. In another study [7], it was shown that wild-type isolates can be selected for earlier reproduction after 47 generations of evolution as well as a generalized decoupling of lifespan and reproductive capacity.

In this paper, it is predicted that mutant genotypes will also produce differences in fecundity when subject to natural selection. For this set of experiments, three classes of mutant genotype with potential effects on fecundity and reproduction have been chosen: AMPK, *daf* and AMPK/*daf*. During times of stress, AMPK mutants exhibit defects in metabolism, which can impact germline development and hence reproductive capacity [8]. In the case of *daf* mutants, various mutations in pathways control the transition to a *dauer* phenotype (a polyphenic stress response) in larval development [9]. Depending on the mutation, this can affect both the timing of reproduction and overall reproductive capacity. The AMPK/*daf* mutants will exhibit both of these phenotypes, and perhaps show an even more pronounced effect as the effects of these mutations converge upon the IGF-1 pathway [10]. By imposing natural selection on fecundity for these classes of mutant, we will be able to determine whether selection for fecundity leads to increases in fecundity, or whether selection for fecundity actually leads to selecting for aspects of the stress response.

One way to compare fecundity for two generations that are adjacent in time is to calculate the amount of RCO. As a mathematical model, RCO is a first-order derivative of the population size genealogies. This allows for an evaluation of demographic variation from generation to generation. When the RCO value consistently moves in one direction (e.g. positive or negative values), we can conclude that natural selection is having an effect on fecundity. The concept of carry-over has been used to investigate the immediate effects of ecological conditions on fecundity in birds [11], mammals [12] and the effects of natural selection on fecundity in nematodes [13]. As traditionally defined, RCO characterizes the effects of ecological or life-history variability with differences in fitness observed within populations [14]. In the avian and mammalian literature, biological carry-over is a general effect of experience and history on reproductive fitness observed over a finite time scale [15]. In nematodes, both Diaz & Viney [13] and this paper treat RCO as being restricted to intergenerational evolutionary time scales. By further restricting this to strains of genetic mutant, we can examine the carry-over effects of natural selection due to specific genetic backgrounds.

The ability to impose positive selection for fecundity on populations over relatively short timespans also suggests that experimental evolution can uncover new pathways to the evolution of complex traits. This includes influencing the evolvability and robustness of the genealogies that result from sustained natural selection. In [16], the nematode species *C. remanei* underwent natural selection for heat shock resistance over 10 generations. This form of environmental selection results in negative selection for robustness to heat shock. A less robust response meant that descendent generations were much less resistant to heat shock than non-selected organisms. Yet selection may not be the key driver in such interactions. According to constructive neutral theory [17], mutation is the primary driver of constraints, compensatory functions and novelty, while selection acts merely to filter this variation in various ways. We can observe this to some extent in the form of restoring fitness advantages lost to mutational drift. For example, compensatory mutations play a role in restoring fitness among individual worms in mutation–accumulation lines of *C. elegans* [18] when being derived from large populations and undergoing natural selection [19].

## 2. Results

This analysis will focus on addressing the three research questions. Analysing the effects of natural selection and mutational diversity on fecundity over a finite interval (Question 1) will lead to an assessment of whether or not observed differences in population size are informative with respect to genotypic identity (Question 2). To understand the subtleties of this relationship, an analysis of changes in reproductive timing resulting from positive selection for fecundity (Question 3) is then presented.

**Figure 1.**
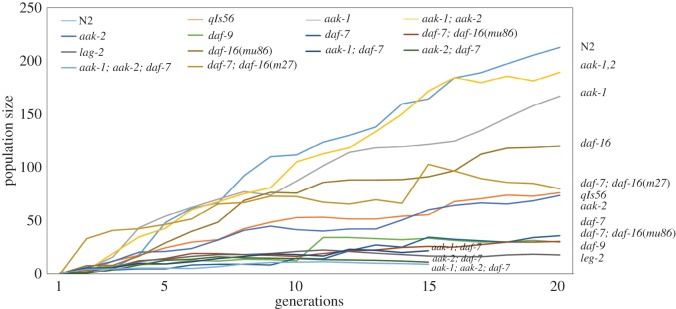
Evolutionary trajectory (measured using mean population size per generation) for 11 strains over 20 generations and 3 strains over 15 generations, normalized using exponential smoothing (*α* = 0.1).

The first question involves examining the effects of selection and mutational diversity on fecundity. To do this, we will first establish the mean level of fecundity within specific genetic strains. Then, we will demonstrate the effects of selection for fecundity by comparing mean population sizes across strains. Using a statistical analysis called exponential smoothing (figure 1), we can derive a population mean per generation for 14 strains described in table 1 that removes many of the demographic fluctuations observed in the raw data (figure 1).

**Figure 2.**
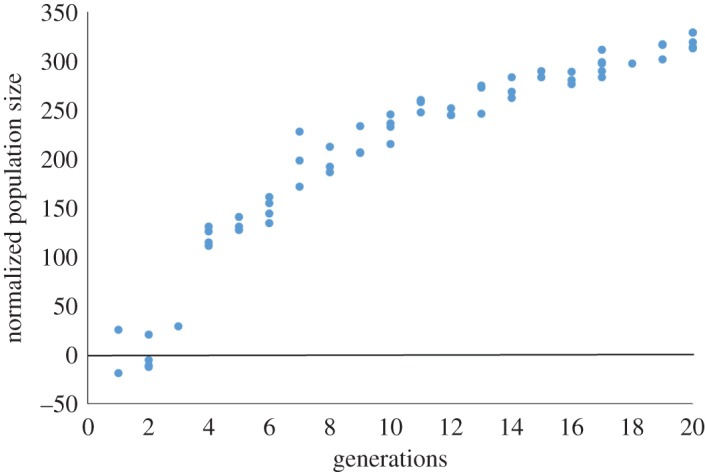
Twenty generations of experimental evolution on the wild-type (N2) strain (62 genealogies). Normalized population size is a non-evolved control subtracted from the measured population size.

**Figure 3.**
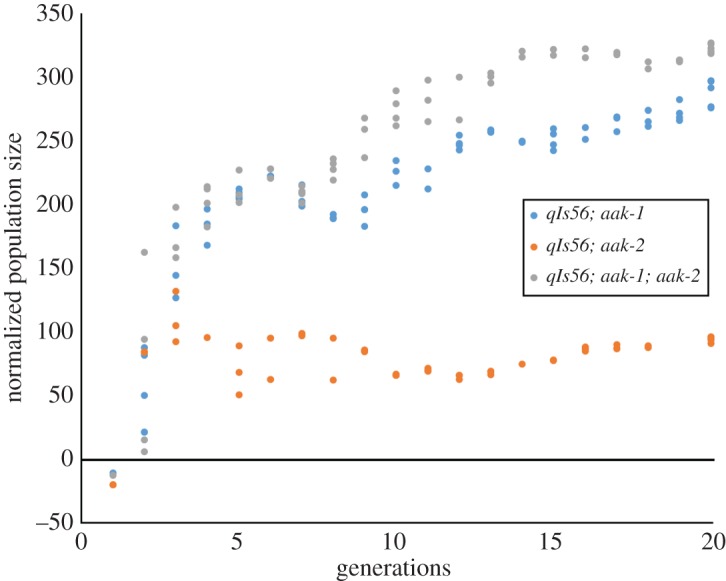
Twenty generations of experimental evolution on the AMPK mutant strains (62 genealogies for *aak-1*, 50 genealogies for *aak-2* and 58 genealogies for *aak-1; aak-2*). Normalized population size is a non-evolved control subtracted from the measured population size.

**Figure 4.**
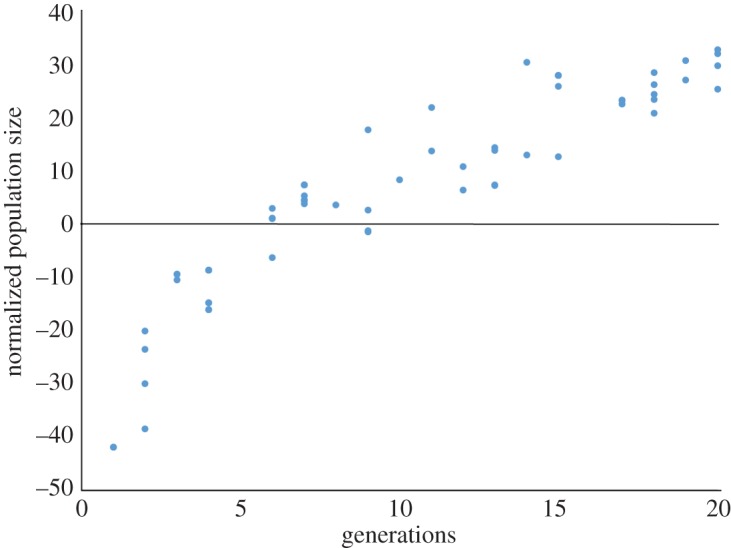
Twenty generations of experimental evolution on the *daf-7(e1372)* strain (62 genealogies). Normalized population size is a non-evolved control subtracted from the measured population size.

**Figure 5.**
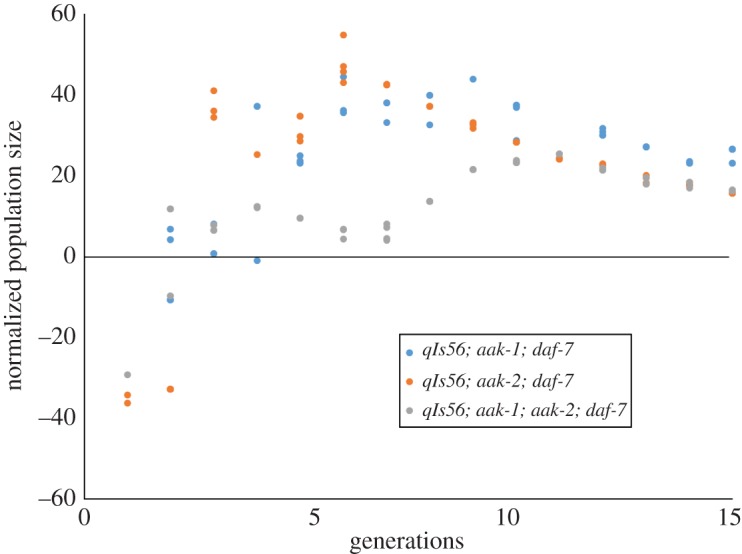
Fifteen generations of experimental evolution on the AMPK/*daf-c* mutant strains (47 genealogies for *aak-1; daf-7*, 51 genealogies for *aak-2; daf-7* and 53 genealogies for *aak-1; aak-2; daf-7*). Normalized population size is a non-evolved control subtracted from the measured population size.

According to the analysis in figure 1, we should expect the wild-type (N2) to exhibit the greatest increase in fecundity, followed by the two AMPK mutants *aak-1(tm1944)* and *aak-1(tm1944); aak-2(ok524)*, and then the *daf-16(mu86)* mutant. Note that the *daf-c* and *lag* mutants exhibit the least pronounced gains in fecundity. In figures 2–5, we will look at a slightly different characterization of mean population size (mean population size for every genealogy) to examine fitness gains relative to fecundity.

We can also examine fecundity gains by comparing the evolved population size with an unevolved population size measurement (see Material and methods, Normalized population size). Figure 2 shows that for the wild-type (N2), there is an initial period (three generations) where the evolved population size is roughly comparable to that of the unevolved population size. However, beginning at Generation 4, there is a jump in normalized population size that progresses logarithmically until Generation 20. While the logarithmic signature is expected due to sampling bias across the length of a genealogy, this demonstrates a signature of positive selection for sustained selective pressure on early fecundity.

Figure 3 shows two sets of outcomes for the AMPK mutants. For the *aak-2(ok524)* mutant, there is a jump in normalized population size at Generation 2, which plateaus at a value of 100 after Generation 10. In the case of the *aak-1(tm1944)* and *aak-1(tm1944); aak-2(ok524)* mutants, there are two jumps in normalized population size before Generation 5. This leads to a logarithmic increase as they approach Generation 20. At Generation 20, both the single *aak-1* and double mutant exhibit normalized population size values comparable to that of N2.

In the case of the *daf-7(e1372)* mutants (figure 4), there is the same logarithmic progression of fecundity observed in figures 2 and 3, but originating at a much lower starting point. As is also the case with figures 2 and 3, the *daf-7(e1372)* mutants exhibit an early spike (at Generation 2) in fecundity. Figure 5 shows the normalized population size over 15 generations for the three AMPK/*daf-7(e1372)* mutant genotypes. As with the AMPK and *daf-7* mutant genotypes described in figures 3 and 4, we observe an early spike in fecundity culminating in peak fecundity during Generation 6 for the single mutants *aak-1(tm1944); daf-7(e1372)* and *aak-2(ok524); daf-7(e1372)*. For longer genealogies, however, we do not see the same logarithmic increase. Instead, we observe a decline in fecundity and intra-strain variation after Generation 10.

**Figure 6.**
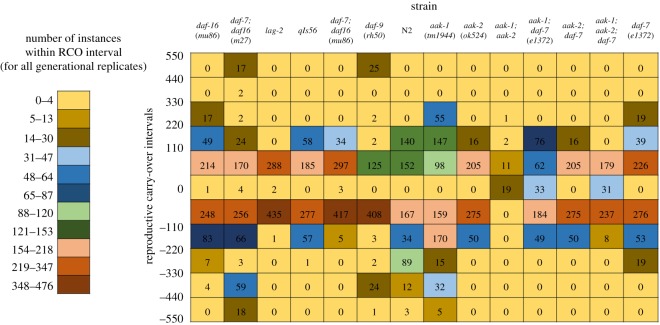
A heat map of reproductive carry-over (RCO) measurement values for 14 strains (13 mutant strains and 1 wild-type) over 15–20 generations. All bins (vertical axis) are of size 110 except for the zero bin. The coloured legend indicates the relative frequency of individual population/generation instances result in a range of RCO values, while the numbers in each cell reveal the exact number of generational replicates that exhibit a value with that range.

The second question can be answered with a visualization of the RCO measure across genetic strains. In wild isogenic lines, Diaz & Viney [13] have observed inter-line differences in mean reproductive variance that are negatively related to mean lifetime fecundity. We can use the RCO measurement to examine trends in fecundity for all generational replicates across all of the evolved strains. Figure 6 shows a heat map for the distribution of RCO values across the evolutionary trajectory for 14 strains.

These data reveal a number of inter-strain differences not directly linked to mutations in specific genes. To further appreciate this, we can compare figures 1 and 6, which demonstrate the smoothed population size over evolutionary time and the distribution of RCO values, respectively. Strains such as *lag-2*, *aak-2* and the AMPK/*daf-7* mutants do not attain large population sizes over the course of their evolutionary trajectory. As a result, they also do not exhibit many negative RCO values. In other strains that never attain large population sizes (*daf-7* and *daf-9*), negative RCO values can be tied to demographic fluctuations (or, more specifically, the downward portion of that fluctuation).

A more subtle effect involves evidence for positive selection on developmental delay. In some strains, as individual worms were positively selected for fecundity, there was corresponding negative selection on developmental timing. For example, in the AMPK/*daf-7* mutants, the 3 day reproductive period that defined a generation was often only enough time for the adult worm to lay eggs. By contrast, in strains such as N2 the 3 day reproductive period that defined a generation produced many L4 stage offspring to select from. This difference can be seen quantitatively in figure 6 in that strains that exhibited this phenomenon also tended to have RCO values for each generational replicate that were strongly negative or exhibit RCO values that range from 150 to −150. While variation between replicates is expected due to stochastic fluctuation, observing less variation within specific mutant strains (mutant genotypes) suggests that the genotype plays a role in depressing fecundity.

**Figure 7.**
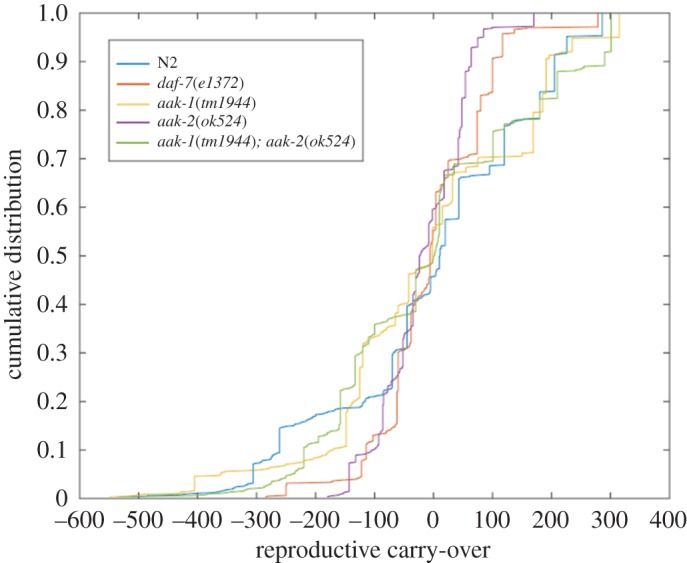
Cumulative distribution functions of the RCO measure for three AMPK mutant genotypes, a *daf-c* mutant (*daf-7*) and the wild-type (N2).

To compare the distribution of RCO values between selected strains in terms of their probability distribution, we plot the cumulative distribution function (CDF) for each strain and compare their shape. This can give us more information about the statistical context of RCO measurements for each strain. Figure 7 shows a comparison between the wild-type (N2), a *daf-c* control (*daf-7*) and three AMPK mutants (*aak-1*, *aak-2* and *aak-1; aak-2*). The wild-type (N2) and *aak-1* strains have similar distributions, as do the *daf-c* control (*daf-7*) and *aak-2*. The double mutant (*aak-1; aak-2*) shares features of both pairs. All strains differ very little around the mean, which is observed also in the heat map.

**Figure 8.**
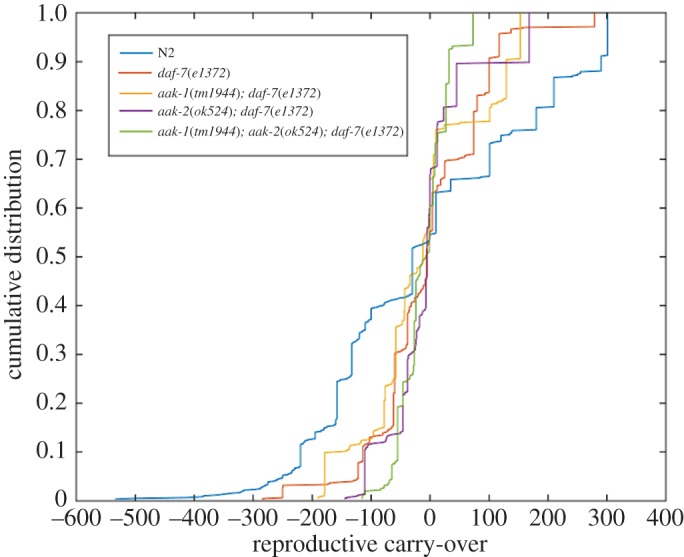
Cumulative distribution functions of the RCO measure for three AMPK/*daf-c* mutants (*aak-1; daf-7*, *aak-2; daf-7* and *aak-1; aak-2; daf-7*), a *daf-c* mutant (*daf-7*) and the wild-type (N2).

Figure 8 shows data for the wild-type (N2) and *daf-c* control (*daf-7*) also shown in figure 7, in addition to the three AMPK/*daf-7* strains (*aak-1; daf-7*, *aak-2; daf-7* and *aak-1; aak-2; daf-7*). In this case, the CDF for N2 is quite distinct from the CDFs for the *daf-7* and AMPK/*daf-7* genetic backgrounds. In particular, N2 differs from the other strains in terms of the tails of its distribution. By contrast, the *aak-2; daf-7* and *aak-1; aak-2; daf-7* strains exhibit extremely short tails, which underscores the lack of variation and overall lack of demographic fluctuation in these genotypes.

**Figure 9.**
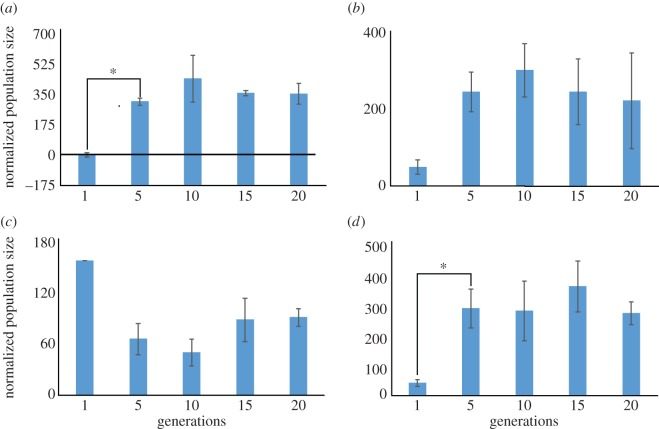
Normalized population size versus five discrete generations of the wild-type (N2) genotype and three AMPK mutant genotypes: N2 (*a*), *aak-1* (*b*), *aak-2* (*c*), *aak-1; aak-2* (*d*). Starred pairs are statistically significant (two-tailed *t*-test).

To demonstrate that there is in fact selection for reproductive timing, we must make a more implicit connection between reproductive potential and the presence of offspring themselves. Our third question is answerable by identifying potential signatures of selection in the evolutionary data. We accomplish this by sampling an evolutionary time series every 4–5 points and comparing population size and its standard error. In figure 9, we can see the normalized population size at five different points within a 20 generation series for the wild-type (N2) and three different AMPK mutant strains (*aak-1(tm1944)*, *aak-2(ok524)*, *aak-1(tm1944); aak-2(ok524)*). These results confirm some of the outcomes observed in figures 1 and 2. For example, in the wild-type (N2) and two mutant genotypes (*aak-1*, *aak-1; aak-2*), there is an early shift towards larger mean population sizes before Generation 5 (figure 9*a*,*b*,*d*).

Based on the results of a Bonferroni-corrected *t*-test, the shifts for N2 and *aak-1; aak-2* between Generations 1 and 5 are statistically significant, *p* < 0.03 and *p* < 0.02, respectively. There seems to be a lack of selection afterward, although the standard errors for the *aak-1* mutant genotype (figure 9*b*) become more pronounced in later generations. In the case of the *aak-2* mutant genotype (figure 9*c*), we see an opposite pattern from the other AMPK mutants. After an initial drop-off in fecundity that is not statistically significant, there appears to be a continued lack of selection. This is distinct from negative selection for fecundity, as the population size measure does not produce any statistically significant decreases.

**Figure 10.**
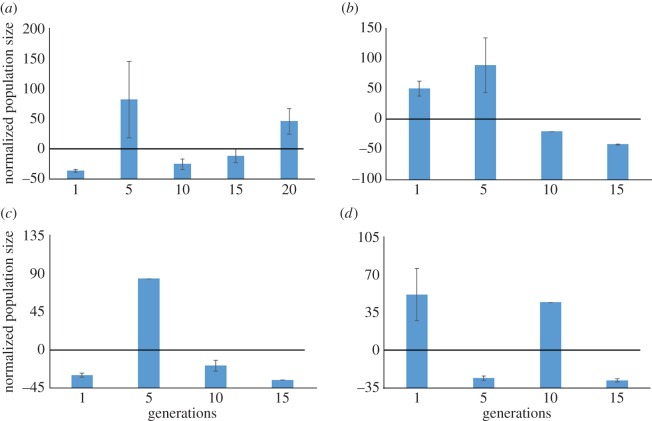
Normalized population size versus five discrete generations of the *daf-7* mutant genotype and three AMPK/*daf-c* mutant genotypes: *daf-7* (*a*), *aak-1; daf-7* (*b*), *aak-2; daf-7* (*c*), *aak-1; aak-2; daf-7* (*d*).

Figure 10 shows a more mixed result. In this comparison, the *daf-7(e1372)* strain is used as a control (figure 10*a*). This shows a large increase normalized population size at Generation 5, accompanied by a drop-off in normalized population size for Generations 10 and 15. Normalized population size increases once again for Generation 20. Conservatively, we can say that this increase is due to demographic fluctuation, and is not due to quick evolutionary changes. This is confirmed by the reproductive behaviour of the AMPK/*daf-7* mutants (figure 10*b–d*), all of which exhibit transient fluctuations. The only strain to show some evidence of negative selection of normalized population size over time is *aak-1(tm1944); daf-7(e1372)*. In this strain (figure 10*b*), normalized population size is above the control in Generation 1 and 5. Somewhere between Generation 5 and Generation 10, normalized population size drops below the control and remains there for the rest of the experiment.

## 3. Study caveats

There are several caveats to the experiments presented here that might impact replicability and/or comparison between the wild-type and various mutant strains. The first of these is the founding population size immediately following a round of selection (every new generation). This is always a single worm regardless of the population size that it is drawn from (1/*n*). While this allows for consistency of method both within and between strains, it may also introduce an additional stochastic component affecting the population size time series. As only a maximum of five worms can be drawn from a single population, we are dealing with large degree of genetic drift in each genealogy generated. In fact, we may potentially lose hundreds if not thousands of novel mutations generated over the course of a single 20 generation experiment.

A more prosaic concern is the nature of the bacterial lawn that provided energy to the reproducing worms. While diet is a key force of natural selection, accounting for variation in diet across replicates, generations and strains is a challenge. While the amount of bacteria is kept constant throughout the collection of experimental and control data, the fact that it was not UV irradiated or constituted as a defined media may have resulted in stochastic fluctuations measured as variability in population size due to diet variability across the course of the experiment. The use of raw OP50 may have also led to small amounts of contamination, which may have retarded egg-laying and otherwise affected parental metabolism. The smoothed time series shown in figure 1 allows us to filter out those potential confounds. Yet the worst-case scenario suggests that larvae selected under the fitness criterion would be robust to the media conditions rather than products of the most fecund parent. If this were a significant factor, it would probably affect the metabolic mutants (AMPK) more than other strains.

## 4. Discussion

Taking these data collectively, we can see that there are two effects of positive selection on fecundity. These differing effects are contingent upon the genetic background of the mutant strain in question. One effect is positive selection on reproductive timing, which results in nonlinear population size increases. This is observed in the wild-type, in addition to the AMPK mutants. The alternative effect is negative selection on reproductive timing, which results in no clear signature of selection on population size and minimization of both the smoothed population size and the RCO measure.

We can also see a clear nonlinearity in terms of selective advantage. In a number of strains, there appears to be an early phase and a later phase of evolution. In the early phase, we see either a large and unambiguous jump in fecundity along with large variability in the RCO. Characteristics of later evolution include logarithmic and convergent behaviour, while changes in population size due to positive selection for fecundity become less pronounced. This two-phase evolutionary trajectory does not seem to be strain-dependent, and varies from strain to strain only in terms of its absolute timing and magnitude.

The effects of positive selection on fecundity appear to be strain-dependent. For our wild-type strain, positive selection did indeed lead to greater fecundity. In this case, selecting for larger brood sizes did lead to a speed-up in reproductive timing whereby larger brood sizes were maintained. However, in the case of our *daf-c* and AMPK/*daf-c* mutant strains, positive selection for greater fecundity actually led to smaller brood sizes over our generational interval. This may be due to some form of developmental/reproductive delay [20], which in the case of our AMPK/*daf-c* mutant strains drives our genealogies to premature extinction.

The AMPK mutants used in this paper have the potential to exhibit extensive reproductive defects when exposed to developmental stress. This leads to a number of results analogous to what is shown in figure 7. For example, in other experiments conducted with the same set of genotypes [20,21], the single mutant *aak-1* and double mutant *aak-1; aak-2* respond differently to extended L1 arrest (where the organism is deprived of a food source for several days, thus extending the L1 larval stage) than do *aak-2*. The smaller effect in the double mutant suggests that the *aak-2* mutant allele has a larger deleterious effect in the face of stress, and the double mutation exhibits a compensatory effect [22].

If we keep this result in mind when making sense of selection for fecundity over several generations, it might suggest that when the *aak-2* mutant is selected for fecundity, it produces smaller populations (and notably smaller fluctuations in the RCO value) relative to the other AMPK mutants (*aak-1* and *aak-1; aak-2*). According to this interpretation, this would also hold true for the N2 (wild-type) genotype, as the wild-type genotype exhibits no potential for reproductive or metabolic defects. Indeed, figure 7 shows that the N2, *aak-1* and *aak-1; aak-2* genotypes all exhibit both larger smaller and larger negative RCO values than observed in the *aak-2* mutant strain.

Both the dual effects of positive selection and two-phased evolutionary trajectory observed in these experiments can be explained in terms of fitness landscape dynamics. An empirical fitness landscape, or genotype-fitness map [23], provides us with a surface to characterize both fitness gains (hill-climbing) and losses (valley descent). In terms of characterizing trends in population size among our genealogies, populations representing those strains that demonstrate a clear logarithmic increase in fecundity over evolutionary time have climbed towards a fitness peak. By contrast, populations representing strains that demonstrate fluctuation in fecundity over evolutionary time are travelling the neutral parts of the fitness landscape and even exploring local fitness minima. For some genetic backgrounds, selecting for fecundity is not particularly advantageous from both a developmental and reproductive standpoint. This is consistent with Stoltzfus [24], who argues that an evolving population evolves towards a fitness peak via mutational diversity rather than the optimization of traits.

The question remains as to what could be driving these processes at the genetic level. Recall that we observed generalized patterns that do not directly correspond to mutations on specific genes or gene families. This suggests that the products of selection are due to antagonistic pleiotropy [25] and other complex interactions [26]. Even though we are studying hermaphroditic genetic mutant lines subject to extensive backcrossing, we are still generating thousands of random mutations per strain. While these mutations may be neutral and occur at very low frequencies, they may also contribute to individual variation and greater evolvability. For a better understanding of this, we need to link systematic gains and losses in fecundity to their potential life-history mechanisms. One the one hand, some mutant genotypes recapitulate the fitness maximization patterns of the wild-type, albeit at a lower order of magnitude. In the case of our AMPK mutants, this effect is due to the characterized function of these mutants, which is not a developmental defect *per se*, but rather a physiological defect which may impair their reproductive capacity. On the other hand, the *lag* and *daf-c* mutants exhibit specific defects in developmental processes. While the functions of these mutants are not directly tied to changes in the heterochronic timing [27,28] of reproduction, they might nevertheless act against fitness gains that would otherwise result from positive selection. This latter point is consistent with Lang & Desai [29] who argue that experimental evolution tends to produce fitness increases via epistasis and parallel pathways associated with diverse sets of mutations.

## 5. Material and methods

### 5.1. Organisms

#### 5.1.1. Strains

Seven strains were acquired from either the Caenorhabditis Genomics Center (CGC, http://cbs.umn.edu/cgc/) or Nathan Schroeder's Laboratory at University of Illinois Urbana-Champaign. These genotypes were: *qIs56[Plag-2::GFP]; daf-7(e1372)*, *qIs56[Plag-2::GFP]; daf-9(rh50)*, N2, *myIs14[Pklp-6::GFP]; daf-7(e1372); daf-16(mu86)*, *qIs56[Plag-2::GFP], lag-2(q420)*, and *daf-16(mu86)*. Further information regarding the [Plag-2::GFP] and [Pklp-6::GFP] constructs can be found in [30]. Strains were grown at 15°C, 22°C and 25°C, depending on the strain (table 2).

**Table 2.** A list of the strains, temperature at which each strain was raised and generation length in days.

strain (genotype identification)	temp. raised (°C)	generation length (days)
*qIs56[Plag-2::GFP]; daf-7(e1372)*	22	6
*qIs56[Plag-2::GFP]; daf-9(rh50)*	22	6
N2	25	3
*qIs56[Plag-2::GFP]; aak-1(tm1944)*	25	3
*myIs14[Pklp-6::GFP]; daf-7(e1372); daf-16(mu86)*	25	3
*qIs56[Plag-2::GFP]*	25	3
*lag-2(q420)*	15	6
*qIs56[Plag-2::GFP]; aak-2(ok524)*	25	3
*qIs56[Plag-2::GFP]; aak-1(tm1944); aak-2(ok524)*	25	3
*qIs56[Plag-2::GFP]; daf-7(e1372); daf-16(m27)*	22	3
*qIs56[Plag-2::GFP]; aak-1(tm1944); daf-7(e1372)*	22	6
*qIs56[Plag-2::GFP]; aak-2(ok524); daf-7(e1372)*	22	6
*qIs56[Plag-2::GFP]; aak-1(tm1944); aak-2(ok524); daf-7(e1372)*	22	6
*daf-16(mu86)*	25	3

#### 5.1.2. Genetic crosses

Seven additional crosses were constructed to gain additional data about the contributions of the AMPK and *daf-7* mutant genotypes to evolvability and developmental plasticity. These genotypes were: *qIs56[Plag-2::GFP]; daf-7(e1372)*; *daf-16(m27), qIs56[Plag-2::GFP]*; *aak-1(tm1944); daf-7(e1372)*, *qIs56[Plag-2::GFP]; aak-2(ok524); daf-7(e1372)*, *qIs56[Plag-2::GFP]; aak-1(tm1944); aak-2(ok524); daf-7(e1372)*, *qIs56[Plag-2::GFP]; aak-1(tm1944), qIs56[Plag-2::GFP]; aak-2(ok524)*, *qIs56[Plag-2::GFP]; aak-1(tm1944); aak-2(ok524)*. More details on the design can be found in the Dryad data repository at http://dx.doi.org/10.5061/dryad.c96md.

### 5.2. Measures

#### 5.2.1. Exponential smoothing

Exponential smoothing is used to analyse the population size time series by removing the noise of demographic fluctuation and setting the initial condition of the smoothed Generation 1 to a value of 1. The exponential smoothing kernel is applied recursively and defined as

5.1 $$F_{t}=\alpha D_{t}+(1-\alpha )F_{t-1},$$

where *F_t_* is the current point in the smoothed evolutionary trajectory, *D_t_* is the corresponding timepoint in the unsmoothed time series (population size), *F_t_*_−1_ is the previous point in the smoothed evolutionary trajectory and *α* is the smoothing constant.

#### 5.2.2. Reproductive carry-over

RCO is a discrete, subtractive first-order time derivative of the population size. RCO can be calculated recursively on an averaged time series, or on individual genealogies. RCO is formally stated as

5.2 $$\text{RCO}=(P_{i})g_{t}-(P_{i})g_{t-1},$$

where *P_i_* are populations derived from the same founder worm, *g_t_* is the current generation in the genealogy and *g_t_*_−1_ is the previous generation in the genealogy. This results in 19 timepoints for an evolutionary trajectory of 20 generations.

#### 5.2.3. Normalized population size

Normalized population size is calculated by subtracting a control population size (generated from an independent set of populations grown under identical environmental conditions) from the single genealogy or population average for each of the 15–20 generations (depending on the strain). Control populations are generated using a modified form of the hanging drop protocol. Normalized population size is calculated for every generation in the evolutionary trajectory against a constant value for the control population.

### 5.3. Statistics and analyses

#### 5.3.1. Graphs

All graphs and data presentation were made in Excel, Matlab and R. All statistical analyses were run in Matlab and R.

### 5.4. Techniques

#### 5.4.1. Determining population size

For every generation, five replicates were created. After a 60 mm diameter plate filled with NGM agar is seeded with 100 µl of OP50 media and allowed to dry, a single L4 stage worm is placed on each of the five plates. Each plate is allowed to grow for 3 days at optimal temperatures for the given strain. At the end of the 3 day period, plates are examined for offspring and counted. Anywhere from 1 to 3 plates then are selected to represent the subsequent generation. Five new plates are seeded from the selected populations with L4 stage representatives in proportion to their population size (e.g. larger populations get more representatives in the next generation).

#### 5.4.2. Selection criterion

As discussed in the population size section, all populations were selected for fecundity at 3 days after being derived from L4 stage individuals. In cases where no offspring were produced, the window for selection was increased to 6 days based on observations of a delayed life-history (table 2). Populations were selected on the basis of fecundity and their population size relative to the other replicate populations in that observation. Therefore, the selection criterion was not absolute, but was consistently applied across all strains.

#### 5.4.3. Hanging drop method

The hanging drop method was proposed in [31] as a means of assessing the fecundity of a single worm over a discrete period of time isolated from maternal effects and with minimal counting error. In this paper, a modified version of this method is used to determine a control population size independent of the evolved populations. Normalized population size measurements were calculated for every generation using a randomly selected set of controls, which were produced using the modified hanging drop method.

The modified hanging drop method is conducted by harvesting a series of worms at the L4 stage and distributing one worm per 60 mm diameter plate filled with NGM agar, which was seeded with 100 µl of OP50 media. Single-worm isolation is accomplished by placing each worm on the agar surface (as opposed to the lid as done in [31]). Every 24 h, each plate (each containing a single worm) is checked for offspring. The number of offspring is counted, and the parent is transferred to a new plate. This was done over the course of 3 days for each evolved strain to serve as a control. The population count for each strain was derived by summing all three daily measurements per replicate and then averaging across the replicates. These measurements were not obtained on ‘Generation 0’ worms, but rather on randomly selected worms of the same genetic strain from the same stock. This was done to obtain a measurement of typical fecundity across the genetic strain rather than amplifying potential instances of individual variation [32].

#### 5.4.4. Genotyping for mutant construction

Genotyping for the *aak-1(tm1944)* and *aak-2(ok524)* mutants was done using the following primers: *tm1944*; internal (TCACACGTCTCTTCCGTGTT), left flanking (TCGCGTCCAGAAGAAGATTT), right flanking (TCCCTTTCTTCGCTCACTTT). *ok524*; internal (CAAAGTCCGCAATCTTCACA), left flanking (TCATCCGCCTCTACCAAGTC), right flanking (TCAAATCCCATTTCGCTTTC). Sequences for primer design were retrieved from GenBank http://www.ncbi.nlm.nih.gov/. Primer design was conducted using Primer3 (http://biotools.umassmed.edu/primer3/primer3web_input.htm). Using Blastn, primers were evaluated (e.g. *E*-value) for similarity with bacterial sequences and other nematode sequences.

## Supplementary Material

Supplemental-File-1

## Supplementary Material

Supplemental-File-2

## Data accessibility

All primary data (genealogies, control conditions) and calculations for selected measures can be found in the Dryad data repository in a tab-delimited (Excel) format: http://dx.doi.org/10.5061/dryad.c96md [33].
