# Supplementary material for: Evolution in eggs and phases: experimental evolution of fecundity and reproductive timing in Caenorhabditis elegans
Source: R Soc Open Sci. 2016 Nov 9;3(11):160496. doi: 10.1098/rsos.160496 (PMC5180133; doi:10.1098/rsos.160496)
Supplement: Supplemental-File-2 [file rsos160496supp2.pptx]

## Slide 1
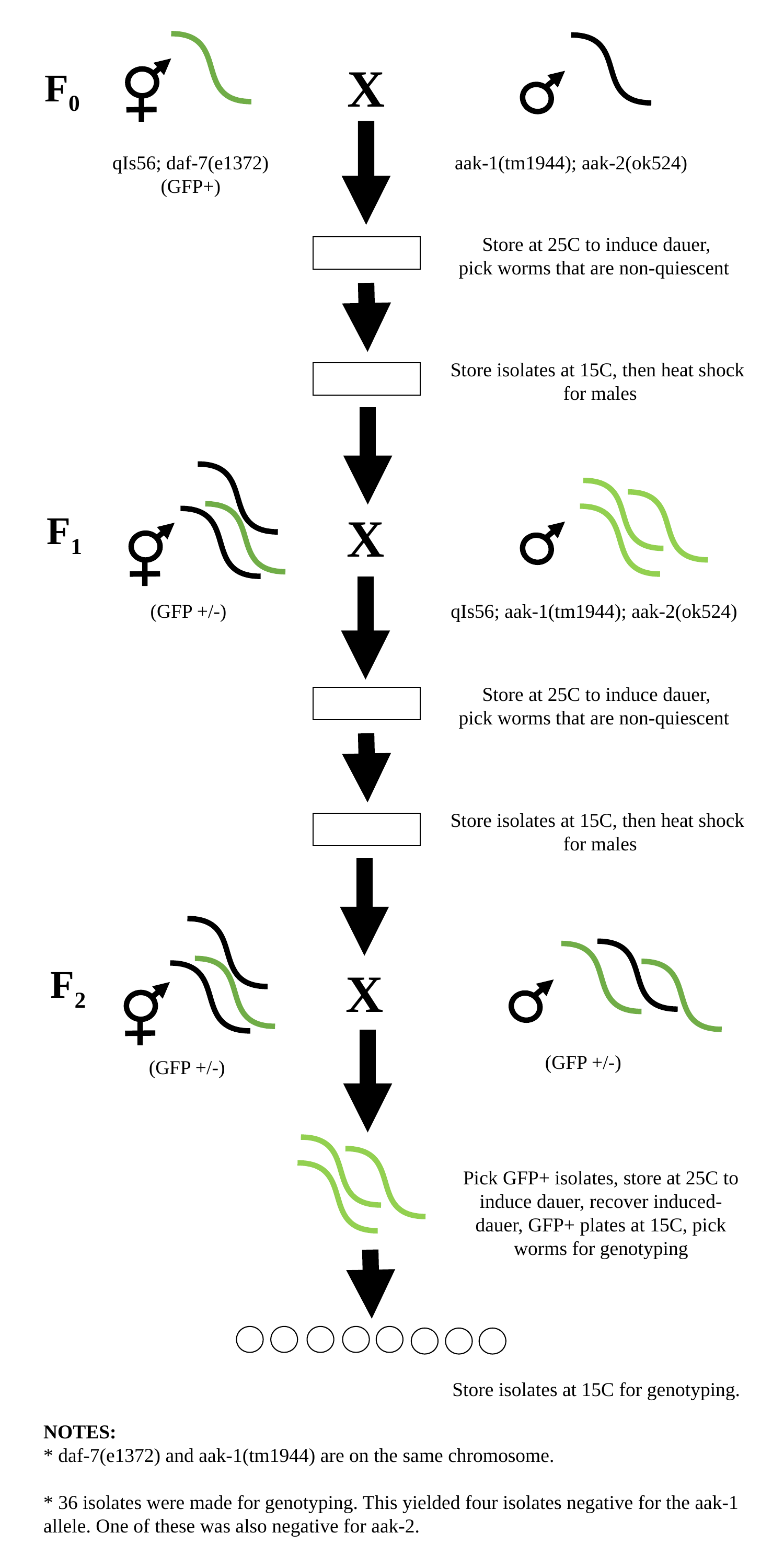

X
F0
aak-1(tm1944); aak-2(ok524)
qIs56; daf-7(e1372)
(GFP+)
Store at 25C to induce dauer,
pick worms that are non-quiescent
Store isolates at 15C, then heat shock
for males
X
F1
(GFP +/-)
qIs56; aak-1(tm1944); aak-2(ok524)
Store at 25C to induce dauer,
pick worms that are non-quiescent
Store isolates at 15C, then heat shock
for males
F2
X
(GFP +/-)
(GFP +/-)
Pick GFP+ isolates, store at 25C to induce dauer, recover induced-dauer, GFP+ plates at 15C, pick worms for genotyping
Store isolates at 15C for genotyping.
NOTES:
* daf-7(e1372) and aak-1(tm1944) are on the same chromosome.
* 36 isolates were made for genotyping. This yielded four isolates negative for the aak-1
allele. One of these was also negative for aak-2.

## Slide 2
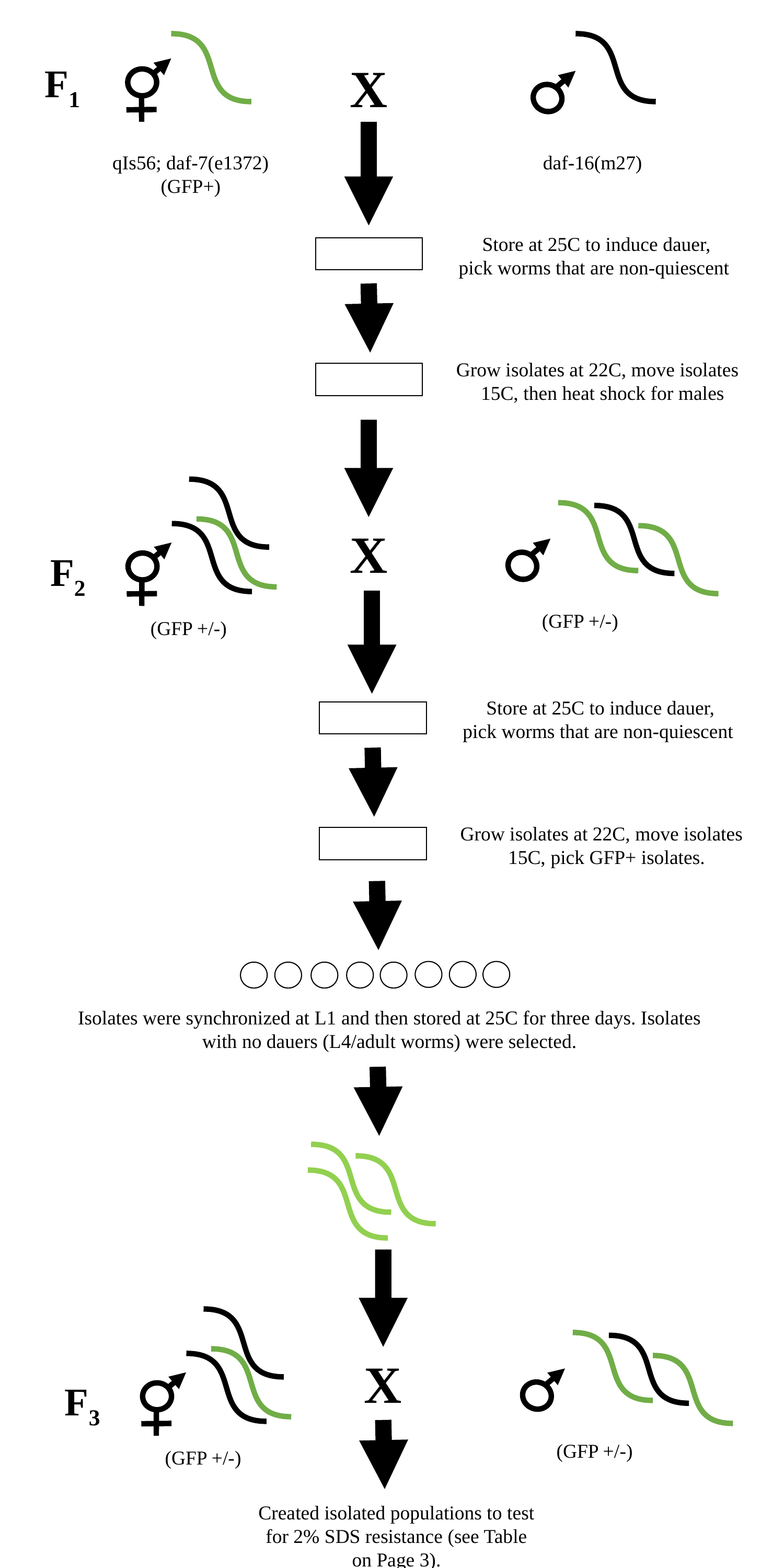

X
F1
daf-16(m27)
qIs56; daf-7(e1372)
(GFP+)
Store at 25C to induce dauer,
pick worms that are non-quiescent
Grow isolates at 22C, move isolates
 15C, then heat shock for males
X
F2
(GFP +/-)
(GFP +/-)
Store at 25C to induce dauer,
pick worms that are non-quiescent
Grow isolates at 22C, move isolates
 15C, pick GFP+ isolates.
Isolates were synchronized at L1 and then stored at 25C for three days. Isolates with no dauers (L4/adult worms) were selected.
X
F3
(GFP +/-)
(GFP +/-)
Created isolated populations to test for 2% SDS resistance (see Table on Page 3).

## Slide 3
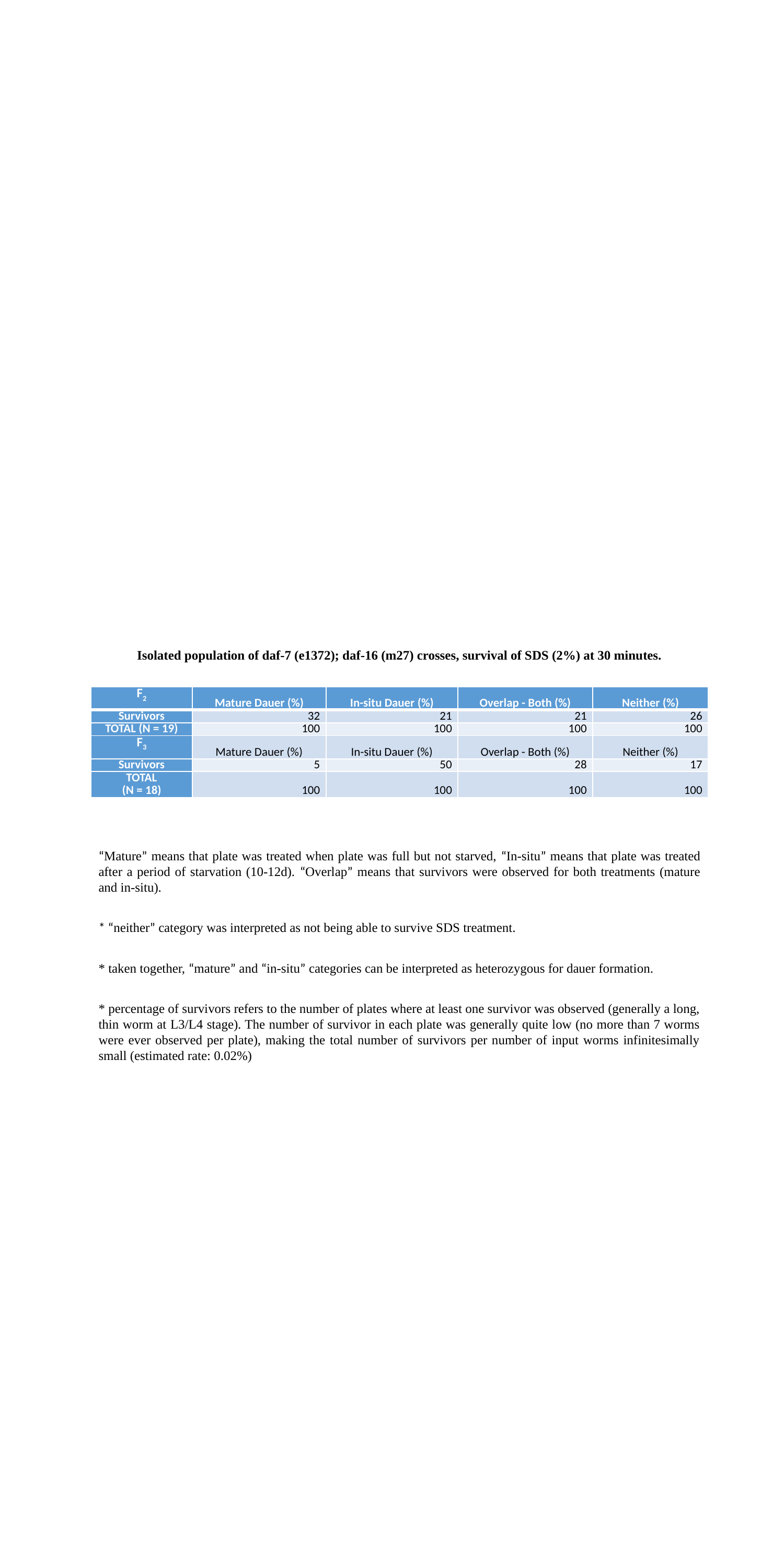

Isolated population of daf-7 (e1372); daf-16 (m27) crosses, survival of SDS (2%) at 30 minutes.
“Mature” means that plate was treated when plate was full but not starved, “In-situ” means that plate was treated after a period of starvation (10-12d). “Overlap” means that survivors were observed for both treatments (mature and in-situ).
* “neither” category was interpreted as not being able to survive SDS treatment.
* taken together, “mature” and “in-situ” categories can be interpreted as heterozygous for dauer formation.
* percentage of survivors refers to the number of plates where at least one survivor was observed (generally a long, thin worm at L3/L4 stage). The number of survivor in each plate was generally quite low (no more than 7 worms were ever observed per plate), making the total number of survivors per number of input worms infinitesimally small (estimated rate: 0.02%)
| F2 | Mature Dauer (%) | In-situ Dauer (%) | Overlap - Both (%) | Neither (%) |
| --- | --- | --- | --- | --- |
| Survivors | 32 | 21 | 21 | 26 |
| TOTAL (N = 19) | 100 | 100 | 100 | 100 |
| F3 | Mature Dauer (%) | In-situ Dauer (%) | Overlap - Both (%) | Neither (%) |
| Survivors | 5 | 50 | 28 | 17 |
| TOTAL (N = 18) | 100 | 100 | 100 | 100 |
